# The Health of Croydon

**Published:** 1901-05-11

**Authors:** 


### THE HEALTH OF CROYDON.

The report for 1900 of Dr. Fegen, the Medical Officer of Health for the Croydon Rural District, discloses a sanitary condition which does infinite credit to his administration, and which enables him to say that the district, while it is fast losing or has even lost its originally rural character and has become of a pronounced urban type, has succeeded in combining all the advantages of a town with none of the disadvantages of the country. What the latter may be Dr. Fegen does not inform us; but he may be presumed to refer to the facilities for locomotion and for the supply of necessaries, in which country districts are unquestionably often deficient. Still, from the point of view of a sanitary officer, the sentence is of a somewhat unusual character, and reminds us of a lady of whom we lately heard, and who, living in the country and writing to her friends in town, declared that she was "pining for a breath of street air." The report enters into considerable detail with regard to the causes of death, and especially with regard to the prevalence of infectious diseases in different parishes; but in these respects there is little to call for remark except a table setting forth the number and distribution of cases of typhoid fever for the past decennium, with a note showing the source of water supply in each locality. The facts thus disclosed appear to lend support to the growing belief that the pollution of water is now, on the whole, effectively guarded against, and that many of the cases of typhoid which still occur must be attributable to the introduction of the microbe through some other channel, such as its conveyance by wind-borne dust. The parishes of Beddington, Morden, Wallington, and Woodmansterne, for example, all derive their water supplies from the Sutton Water Company; and while Beddington and Wallington presented a total of 42 cases of typhoid during the decennium, Morden and Woodmansterne presented a total of 10. Morden escaped entirely during seven years of the decennium, and Woodmansterne during eight; while Beddington escaped only twice, and Wallington only once. A similar difference is noted between Coulsdon and Sanderstead, both supplied by the East Surrey Company; but Merton and Mitchem, both supplied by the Lambeth Company, were alike in suffering severely. With regard to Mitcham, it is said to be regarded as a "tipping ground" for all kinds of refuse from London, and has probably been injured in health accordingly.

### The Census.

It may be regarded as an indication of the existence of a thoroughly healthy state of public opinion on the question of population, that the preliminary Census returns which were issued by the Registrar General on Friday, and which were officially declared to be open to correction if errors were detected in the course of a more complete examination of the papers than had then been possible, were at once challenged by two important boroughs, Chester and Newport, as containing understatements of their respective populations. It is asserted, on behalf of Chester, that the correct figures would show, instead of the alleged decrease of 824, from 37,105 to 36,281, an increase of 5,374, rendering the correct total 42,479; and, on behalf of Newport, that they would show, instead of the alleged increase of only 6,767, from 54,787 to 61,474, a real increase of no less than 12,236, and an actual population of 66,943. How far either of these professed amendments may be correct we have no present means of ascertaining, more especially as, by the provisions of the Census Act, local officials are prohibited from supplying information with reference to their districts; but it is quite clear that, in both the places mentioned, active residents have seen the importance of at once drawing attention to an error which might not only seem to cast some discredit upon the places concerned, but would certainly have a serious influence upon their vital statistics. Time will show to what extent their action will be justified by facts; but it is none the less significant that such action should have been taken, and that no complaint of error in the opposite direction should have been heard from any quarter. For the real lessons to be derived from the Census we shall have to wait for some time longer, since the mere mechanical work of accurate counting and computation cannot be hurried without risk of vitiation of the results; and all we know from the figures issued last week is that 29 metropolitan and 67 county boroughs have added to their aggregate inhabitants about a million and a half in the course of 10 years. With this information, and with the incidental evidence that town populations would regard a diminution in their numbers to be discreditable, we must for the present be content.

### A Medical Man's Obligations.

A decision was lately given in the Southwark County Court which will, we think, considerably astonish many a medical man. The action was one for the recovery of £1 10s. 6d. for professional services. The plaintiff's case was that he attended the defendant's wife and subsequently his child. In regard to his attendance on the child, however, he said that the mother did not carry out his suggestions, and so persistently did things which he had advised her not to do, that, after paying several visits, he wrote declining to attend any more, and suggested that another medical man should be called in. For attendance on the wife his fees amounted to 15s. 6d., and for the child up to the time when he gave up attending 15s. Judgment was entered for 15s. 6d., the amount due for attending upon the wife, but nothing was allowed for attending upon the child, the reason given by the learned judge being that a medical man cannot throw up a case and then charge for his attendance. So far as this particular case goes, there may have been special circumstances of which we know nothing; but in regard to the general principle laid down by the judge we can have not the shadow of a doubt that he is wrong. The plaintiff asked: "But has a doctor no power to retire from a case?" To which his Honour answered: "Yes; but he cannot charge for it. When you are called into a case your obligation is to do your best until you are discharged." And in answer to further questions he said: "You are entitled to say you decline the responsibility, and that you will not attend any more; but you cannot charge for your services. . . . Your engagement was to attend the child during its illness—that is the ordinary engagement in such a case—and if you throw it up you do it at your own risk." It is always difficult to induce lawyers to take the trouble to understand the facts and circumstances of medical work, and this is an instance in point. If one were to engage a man to paint one's house, and he were to "retire" after putting on the first coat, one certainly would decline to pay him for what he had done; and the learned judge seems to have regarded medical attendance from the same point of view, and to have considered that the contract or the implied contract was for "the job." If this were so, no doubt his judgment would be right in law. But it is not so. In ordinary general practice there is no contract, either actual or implied, to attend right through the case. The undertaking is purely and simply to attend at so much a visit so long as it is mutually desired that the attendance shall continue. It is most important that the medical profession should maintain this position. In some cases, it is true, medical "piece-work" is undertaken, as for example in regard to operations and midwifery. In these cases undoubtedly both the payment and the attendance is "by the job," and the contract cannot be broken on either side without forfeit. But equally, undoubtedly, in ordinary general practice, where payment is by the visit, the *status* of the doctor is that of the day labourer, and with reasonable notice on either side he can be dismissed or can give up his engagement. What is reasonable notice will depend partly upon custom and partly upon place. Certainly there is no engagement on either side that the attendance is to continue "during illness"—that is until death or complete restoration. How about attending an old man whose illness never does end until he dies? Is it to be impossible to retire from such a case until one is discharged, without sacrificing all the fees owing, whatever the whims and fancies of such an old man may be? Such a thing only wants suggesting to show the absurdity of the position, which, however, as things now stand, is a judgment of the court.

### London and the Plague.

The arrangements made for dealing with the plague, should we have the misfortune to be attacked by that disease in London, are sufficiently satisfactory so far as they go. It is important, however, that it should be understood that they do not go very far, and that at best they are somewhat of a jumble. It appears that the London County Council and the Metropolitan Asylums Board propose to divide the work between them. Cases of real or suspected plague will be received into the hospitals of the Board and "contact" cases will be watched in places provided by the County Council. On the Council receiving intimation from any medical man, that any person is suffering from a malady raising suspicion of plague, the Council's plague expert will be requested immediately to visit the patient, and he will decide whether or no the patient should be removed to one of the Asylum Board's hospitals. Practically the duty of the practitioner will end when the plague expert comes in. If a few cases of plague should occur from time to time, as is almost sure to be the case, this arrangement will no doubt answer very well. But it is sufficiently obvious that the Asylums Board has no appliances at its disposal for dealing with any extensive outbreak, and that if plague should really attack London seriously, some entirely fresh arrangements would have to be made. In such a case it is to be presumed that the Local Government Board would intervene, and would assume supreme command. In the face of a severe epidemic it would never do to have the control split up between such a number of semi-independent bodies as those which at present are engaged in the work. The proper management of epidemic plague involves much besides the mere removal of cases, and we may feel quite confident that nothing but muddle would result were the work divided between so many authorities; the Asylums Board dealing with those who are attacked, the County Council with those who have been exposed to infection, the parishes with the cleansing, disinfection, and closing of infected houses, and the Local Government Board overriding everything in its own slow and ponderous fashion, and preventing any of the other bodies feeling or indeed possessing any final responsibility to the public for the control of the epidemic. Of all the things wanted were such a catastrophe to occur, the first would be one single head, willing to act at once and to accept responsibility.

### Motors.

We can hardly doubt that the motor-car exhibition which has just been held at the Agricultural Hall will raise hopes in the minds of many medical men of getting rid of some of the troubles of the stable; and in districts where the roads are fairly good we see no reason why these hopes should not soon be realised. As yet, however, the time has hardly come. It is, we think, a misfortune to the whole of the motor industry, and certainly to those who wish to obtain a reliable machine at a reasonable price, that from the first the autocar has been regarded as a pleasure rather than as a business craft. The result of this has been that speed has come to be regarded as the great criterion. No doubt power to maintain a high speed with safety involves workmanship of the highest class, so that in one sense speed does cover all other requirements. Unfortunately the craze for speed has tended greatly to keep up the price of motors. Before long, however, we shall doubtless settle down; the makers will cater for the steady goer who does not want to exceed the law even in England; and then we may fairly hope to see good machines at a lower price than is asked for them at present. What a medical man wants in a motor is not speed. If he can go as quickly as a trotting horse will take him he gets all he wants in that direction. But what he does want is trustworthiness. He wants a machine that can be got ready at short notice, that can be left unattended, perhaps for hours, and can be easily started home again, and above all things he wants one which does not break down. The last is a condition which but few cars comply with. If the makers would but give us a reliable machine we would have no doubt about the motor being the best vehicle for the medical man. Unfortunately, just as a doctor likes to have a good horse, so he does not like to ride a motor of which he is ashamed, and so long as speed remains the criterion doctors, as well as other folk, will waste a good deal of money over motors.

